# The Composition of Gut Microbiota in Patients Bearing Hashimoto's Thyroiditis with Euthyroidism and Hypothyroidism

**DOI:** 10.1155/2020/5036959

**Published:** 2020-11-10

**Authors:** Simo Liu, Yaxin An, Bin Cao, Rongxin Sun, Jing Ke, Dong Zhao

**Affiliations:** ^1^Center for Endocrine Metabolism and Immune Diseases, Beijing Luhe Hospital, Capital Medical University, Beijing 101149, China; ^2^Beijing Key Laboratory of Diabetes Research and Care, Beijing 101149, China

## Abstract

**Aims:**

Hashimoto's thyroiditis (HT), a type of autoimmune disease, occurs due to genetic predisposition and environmental factors. It is well known that thyroid function may affect the gut microbiota. However, the composition of gut microbiota in HT patients with different thyroid function status has been less highlighted. Therefore, we focused on the alterations in the composition of gut microbiota in HT patients with euthyroidism and hypothyroidism.

**Methods:**

We performed a cross-sectional study, including 45 HT patients with euthyroidism, 18 HT patients with hypothyroidism, and 34 healthy controls. Fecal samples were collected, and microbiota was examined by using 16S RNA ribosomal RNA gene sequencing. Then, we analyzed the possible pathways in relation to the enriched bacteria by linear discriminant analysis (LDA) effect size (LEfSe).

**Results:**

Compared with the controls, bacterial richness and diversity were significantly lower in patients with HT, especially in hypothyroidism. Moreover, *Lachnospiraceae_incertae_sedis*, *Lactonifactor*, *Alistipes*, and *Subdoligranulum* were more enriched in HT patients with euthyroidism, while *Phascolarctobacterium* was more abundant in those with hypothyroidism. Further analysis suggested that *Phascolarctobacterium* was negatively related to several pathways, including environmental information processing and metabolism.

**Conclusion:**

In summary, our study demonstrated the altered composition of gut microbiota in HT patients with different thyroid function status. Moreover, *Phascolarctobacterium* may be involved in the development of HT.

## 1. Introduction

Hashimoto's thyroiditis (HT), also termed chronic lymphocytic thyroiditis, is a type of autoimmune disease, affecting approximately 5% of the general population, in particular, the middle-aged females [1]. HT was first described in 1912 by a Japanese surgeon and pathologist, Hakaru Hashimoto [2]. It is characterized by intrathyroidal mononuclear cell infiltration, leading to gradual deterioration of the thyroid gland, and the production of autoantibodies against thyroglobulin (TG) and thyroid peroxidase (TPO) [3]. HT is frequently asymptomatic, with the most common manifestation being an enlargement of the thyroid gland, with or without hypothyroidism. During the course of HT, patients may develop subclinical hypothyroidism, even overt hypothyroidism.

To date, the etiology and mechanism of HT remain unclear. It is extensively accepted that environmental factors play a critical role in the development and progress of HT in genetically susceptible individuals [4, 5]. Several studies have documented that the concordance rate in twins was higher in monozygotic twins than heterozygotic twins [6, 7]. In addition to genetic factors, multiple studies have demonstrated that environmental trigger have been involved in the genesis of HT. Smoking and excessive intake of iodine are important trigger factors [8, 9]. An autoimmune attack is considered as the crucial and final step, which leads to the development of HT.

Human gut microbiota comprises trillions of microorganisms, predominantly bacteria. Human beings and the complicated, dynamic microbial community have co-evolved in a symbiotic relationship [10]. The actions of gut microbiota are not limited to local effects in the gut as 20% of host blood metabolites are derived from commensal bacteria [11]. The vital role of gut microbiota in immunity and metabolism has been well demonstrated [12]. In recent years, numerous studies have shed light on the crucial role of gut microbiota on multiple metabolic and autoimmune diseases, such as diabetes mellitus, lupus, Alzheimer's disease, obesity, and so on [13, 14]. It is well known that the thyroid hormone could influence the gastrointestinal structure and function, especially motility of the gut; meanwhile, a healthy gut microbiota also has beneficial effects on thyroid function [15–17]. Although there are several studies that explore the relationship between gut microbiota and HT, the composition of gut microbiota in HT with different thyroid function status has been less studied. Therefore, we focused on the alteration in gut microbial composition in patients having HT with euthyroidism and hypothyroidism.

## 2. Material and Methods

### 2.1. Ethics Statement

The study was approved by the Ethics Committee of Beijing Luhe Hospital, Capital Medical University, China, and was carried out according to the guidelines of the Declaration of Helsinki. Written informed consents were acquired from each participant.

### 2.2. Study Population and Sample Collection

Ninety-seven female participants, recruited from the outpatient departments of the Center for Metabolic and Immune diseases at the Beijing Luhe Hospital, Capital Medical University, China, were enrolled in our study from June 2018 to March 2019. The study population consisted of 34 healthy volunteers as controls and 63 patients with diagnosed HT. Among the latter group, 45 patients had euthyroidism (HTN), and 18 patients had hypothyroidism (HTH). The diagnosis of HT was defined as follows: (1) elevated serum TPOAb and/or TGAb, and (2) diffused thyroid morphological feature under ultrasound. People with other autoimmune diseases such as Grave's disease, type 1 diabetes, history of thyroid surgeries, and pregnancy were excluded. Patients who used antibiotics in the last one week before collection of fecal samples were also excluded. Venous blood samples were collected from all subjects in the morning following an overnight fast. Fecal samples were collected in the tubes prefilled with fecal DNA-stabilizer, delivered to our laboratory, and stored at –80°C until further processing.

### 2.3. Biochemical Measurements of Thyroid Function and Antibodies

Serum levels of thyroid-stimulating hormone (TSH), free triiodothyronine (FT3), free thyroxine (FT4), total triiodothyronine (TT3), and total thyroxine (TT4) were determined by using chemiluminescent immunoassays (Abbott Diagnostics, Tokyo, Japan). The reference ranges were 0.27–4.2 uIU/mL for TSH, 2.02–4.43 pg/mL for FT3, and 0.93–1.71 ng/dL for FT4. The measurements of serum thyroid autoantibodies (TPOAb or TGAb) were performed by using chemiluminescent immunoassays (Beckman Coulter, Fullerton, CA, USA). The reference ranges for TGAb and TPOAb were defined as less than 115 U/mL and smaller than 34 U/mL, respectively.

### 2.4. DNA Extraction and PCR Amplification

The genomic DNA from fecal samples was isolated by using the sodium dodecyl sulfate lysate (SDS) freeze-thaw method according to the standard protocol. The extracted DNA were soon delivered to Guhe Information Technology Co., Ltd. The integrity of the DNA extracts was examined by agarose gel electrophoresis, and then the DNA samples were diluted to 5 ng/uL using nuclease-free water. The amplification of the hypervariable V4 region of bacterial 16S rRNA gene from genomic DNA was carried out by PCR using universal linkage primers of the Illumina sequencing platform: 515F (5′- GTG CCA GCM GCC GCG GTA A-3′) and 806R (5′- GGA CTA CHV GGG TWT CTA AT-3′). The PCR was carried out using the following conditions: an initial denaturation at 98°C for 30 s, 30 cycles of denaturation for 15 s at 98°C, annealing for 15 s at 58°C, 15 s at 72°C for elongation, followed by a final extension at 72°C for 1 min. The mixture of PCR products with the same volume of 1 × loading buffer was visualized by 2% agarose gel electrophoresis, purified by AXYGEN Gel Extraction Kit (Axygen Biosciences, Union City, CA, USA) and quantified using Qubit 2.0 (Life Technologies, Carlsbad, USA) according to the manufacturer's guidelines. High-throughput sequencing of the 16S rRNA gene in the V4 region was performed using a HiSeq platform (Illumina, San Diego, CA, USA) at Guhe Information Technology Co., Ltd.

### 2.5. Sequence Analysis

Sequencing libraries were developed using NEB Next® Ultra™ DNA Library Prep Kit for Illumina (NEB, USA), and index codes were added. The library quality was assessed on the Qubit@ 2.0 Fluorometer (Thermo Scientific, USA) and the Agilent Bioanalyzer 2100 system. In the end, the library was sequenced on an Illumina HiSeq platform, and 250 bp paired-end reads were generated.

### 2.6. Data Processing

Based on samples' unique barcode, raw reads were assigned to different samples, and then the paired-end reads from the original DNA fragments were merged to raw tags. Clean tags were generated after filtering of the merged raw tags according to specific quality control processes of Vsearch (Version 2.10.4). To obtain effective tags finally, Chimera sequence was detected and discarded by USEARCH (version 10.0.240) using a de_novo method, and the remaining sequence was aligned to Silva database to further discard the chimera sequence. Sequences were assigned to the same operational taxonomic units (OTUs) at 97% sequence similarity. The representative sequences for each OTU were selected and annotated for taxonomic information using Silva database.

### 2.7. Bacterial Diversity Analysis

To assess the microbial diversity within a community (alpha diversity), the OTU table was rarified, and different metrics were calculated based on the genera profile of CON, HTN, and HTH groups, including Richness index and Shannon index. Then, for beta diversity (between-sample), the OTU table was used to generate a matrix, and constrained principal coordinate analysis (CPCoA) was performed and displayed by ggplot2 package in *R* (Version 3.5.2).

### 2.8. Taxonomic Discovery Analysis

Statistically significant differences in the relative abundance of taxa associated with groups were detected by linear discriminant analysis (LDA) effect size (LEfSe). Taxa with linear discriminant analysis (LDA) larger than two at a *P* value < 0.05 were considered significantly enriched.

### 2.9. Functional Metagenome Predictions

Regarding functional metagenome prediction, OTU representative sequences were captured from GreenGenes database by using the USEARCH global alignment command. Reconstruction of the metagenome was performed. Functional genes were predicted by Phylogenetic Investigation of Communities by Reconstruction of Unobserved States (PICRUSt) and were categorized into Kyoto Encyclopedia of Genes and Genome (KEGG) and orthology (KO). In order to explore the relationship between enriched taxa and significant functional metagenomes, Spearman's correlation coefficients were calculated.

### 2.10. Statistical Analysis

One-way analysis of variance (ANOVA) with post hoc Tukey HSD test was conducted to detect differences in metabolic indices as well as the abundance of genera and KOs. *P* values were adjusted in multiple testing followed by the Benjamini–Hochberg correction. A *P* value less than 0.05 was considered significant.

## 3. Results

### 3.1. The Characteristics of the Study Population

All participants (*n* = 97) in the three groups, including healthy controls, HT patients with normal thyroid function (HTN), and HT patients with hypothyroid status (HTH), were females and of Han nationality. The demographic and clinical features of the participants are shown in Table 1. There was no significant difference in BMI among the three groups. Compared with the CON and HTH group, patients in HTH group showed a higher value of mean age (36.3 ± 2.1 vs. 29.6 ± 0.6 and 34.6 ± 1.0, *P* = 0.0003). Among patients with HT, those with hypothyroidism presented a longer disease duration (29.7 ± 10.7 vs. 13.6 ± 2.7, *P* = 0.1600) and a significantly higher proportion of usage of levothyroxine (72% vs. 30%, *P* = 0.0034).

### 3.2. The Gut Microbiota Composition in HT Patients with Euthyroidism and Hypothyroidism

To figure out the difference of gut microbiota in HTN and HTH patients, 97 fecal samples from healthy controls, HTN, and HTH patients were collected and sequenced for the 16S rRNA gene. After data trimming and quality control from a total of 13489147 valid sequences, 11106451 high-quality tags were obtained, generating an average of 105776 sequences for each sample. Subsequently, we acquired 16657 OTUs at a 97% sequence similarity.

**Figure 1:**
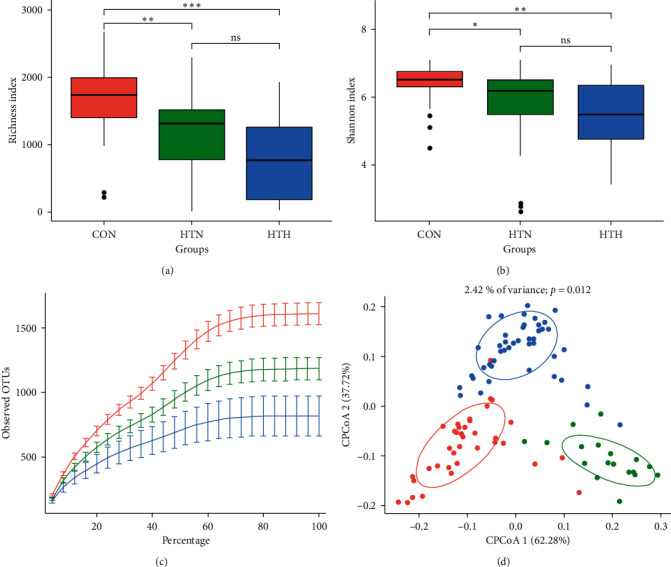
The gut microbiota composition in healthy controls and HT patients with euthyroidism and hypothyroidism. Alpha diversity, including Richness index (a) and Shannon index (b). Rarefaction curves (c) and constrained principal coordinate analysis (CPCoA) (d) according to weighted Unifrac distances were carried out to assess beta diversity among controls (denoted as red circles), HT with euthyroid HT patients (green circles), and hypothyroidism (circles in blue). Ellipses correspond to the clustering of fecal samples for each group encircling 95% confidence interval. CON, healthy controls; HTN, patients bearing Hashimoto's thyroiditis with euthyroidism; HTH, HT patients with hypothyroidism.

Richness index (Figure 1(a)) and Shannon index (Figure 1(b)) demonstrated that patients with HT had a more reduced richness in gut microbiota diversity than healthy controls. In addition, gut microbial richness and diversity were lower in patients with HTH than those with HTN, although the difference was not significant. Rarefaction curves of observed OTUs among the three groups showed the same pattern as Richness index and Shannon index (Figure 1(d)).

Concerning beta diversity, namely, similarity or dissimilarity between bacterial communities of different samples, a plot of CPCoA was analyzed according to the bray distance, demonstrating a distinct separation (*P* = 0.012) for the three groups (Figure 1(d)). These results suggested that the gut microbiota community was different among the three groups, with less richness in bacterial diversity in the HTH group.

### 3.3. The Relative Abundance of Specific Bacterial Taxa Associated with HT

**Figure 2:**
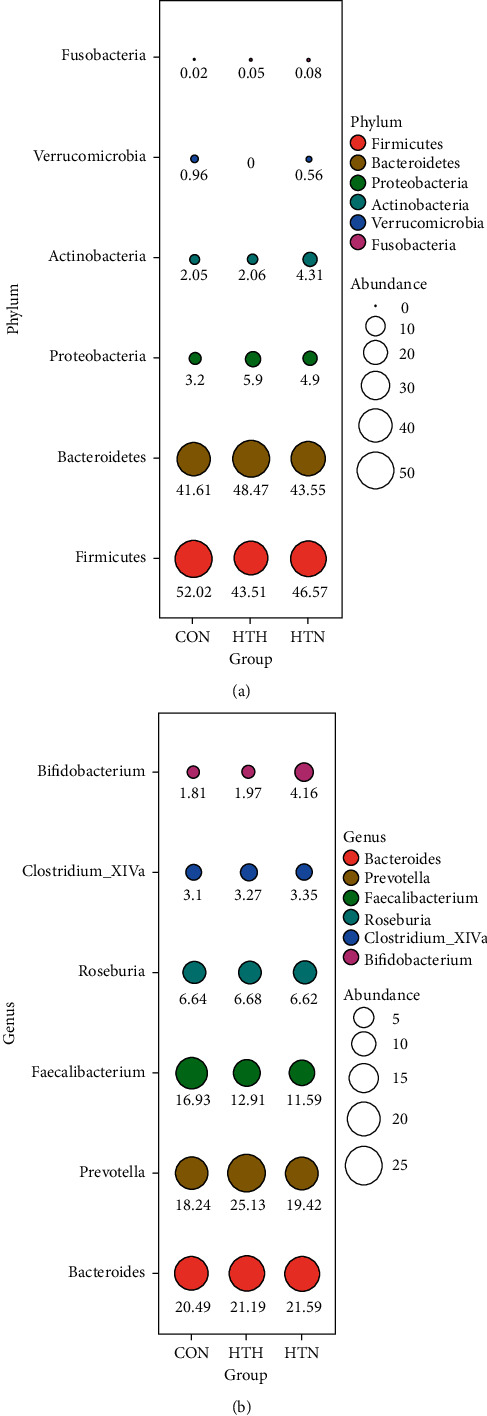
Differences in bacterial taxa abundance among the three groups at phylum and genus levels. The relative abundance of major OTUs expressed in different colors among the three groups was indicated by bubble plot, where the abundance is scaled by bubble area and indicated by number in percentage.

**Figure 3:**
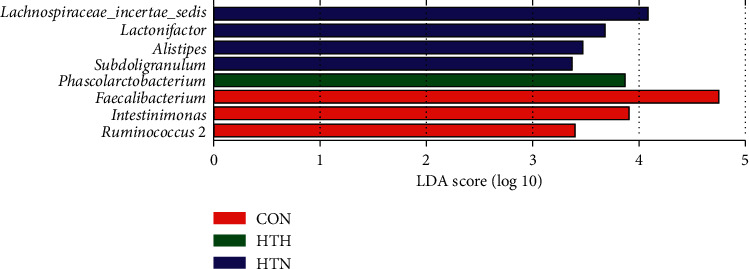
The abundant microbial taxa of fecal community from the three groups. Linear discriminant analysis (LDA) coupled with effect size measurements identified enriched bacterial taxa at the genus level in HTN (blue histograms), HTH (green histogram), and CON (red histograms) group.

Further analysis found the top six bacterial taxa according to the relative abundance at the phylum level (Figure 2(a)) and the genus level (Figure 2(b)). The results demonstrated that healthy controls and patients with HTN shared a more similar pattern in bacterial taxa whether at the phylum or the genus level. More specifically, patients with HTH had significantly higher microbial counts of *Bacteroidetes* in phylum and *prevotella* in genus. Furthermore, LEfSe was used to determine the specific bacterial taxa associated with patients bearing HT of different thyroid function. The data showed that *Lachnospiraceae_incertae_sedis*, *Lactonifactor*, *Alistipes*, and *Subdoligranulum* were more enriched in the HTN group, while *Phascolarctobacterium* was more abundant in those with HTH. In healthy controls, *Faecalibacterium*, *Intestinimonas*, and *Ruminococcus* were more increased (Figure 3).

### 3.4. The Enrichment of Gut Microbiota in Cellular Pathways

**Figure 4:**
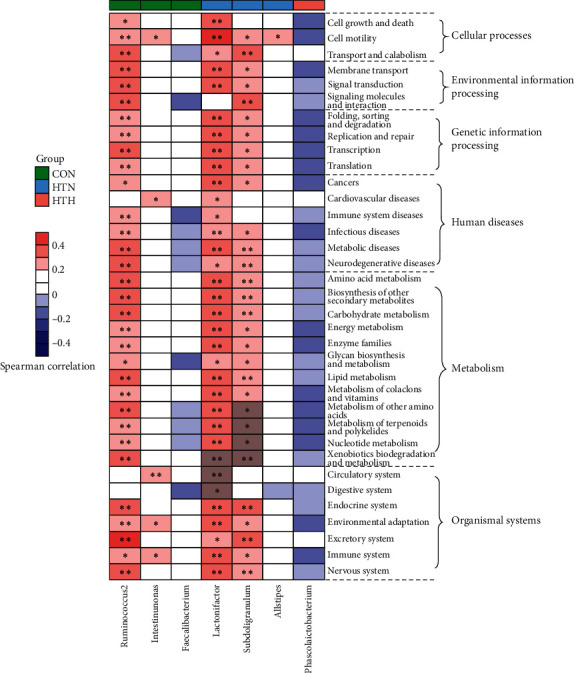
Heatmap of spearman's correlation between enriched bacterial taxa and KEGG pathways according to clustering of three groups. Spearman's correlation coefficients were calculated and are shown in the left, with red representing a more positive and blue being more negative correlation. The clustering of the control group (green box), HTN group (blue box), and HTH (red box) are presented. ^*∗*^Spearman's correlation is significant with *P* < 0.05.

**Figure 5:**
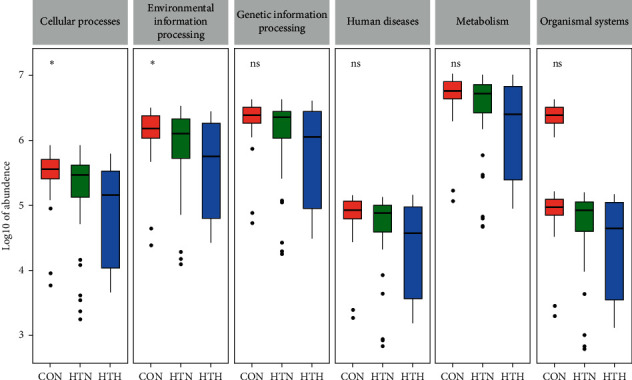
The possible pathways related to gut microbiota change among the three groups. The abundance of predicted genes in each predicted pathway is indicated by boxplot. ^*∗*^*P* < 0.05 indicates significant.

Then we explored the relationship between cellular pathways and different gut microbiota composition. We found that *Phascolarctobacterium*, which was enriched in the HTH group, was involved in negative regulation on most pathways such as cellular process, environmental information processing, genetic information processing, and metabolism. In contrast, except for Faecalibacterium, the other bacteria, enriched in the control and HTN group, were positively correlated to the above pathways (Figure 4). Finally, we investigated the enrichment of gut microbiota in pathways among the three groups. The results suggested that patients with HT, especially those with HTH, were significantly less enriched in bacteria-related cellular processes and environmental information processing pathways (Figure 5). Therefore, we supposed that gut microbiota may be involved in the development and progress of HT, while further studies were still needed to clarify the underlying mechanism.

## 4. Discussion

The association between gut microbiota and autoimmune thyroid diseases (AITD) has become a very popular field in recent years. Many scientists aimed to illuminate the relationship and the underlying mechanism linking gut microbiota and AITD, including Graves' disease (GD) and HT, expecting to find a novel target to prevent the progress of the diseases or reverse the disordered immune system. However, most of the studies in this area focused on the GD and Graves ophthalmopathy (GO) [18–22], while only a few studies have explored the relationship between gut microbiota and HT.

In our study, we investigated the gut microbiota composition in HT patients with euthyroidism and hypothyroidism, showing that the microbial richness of gut microbiota in HT patients was significantly lower than in the control group. Moreover, HT patients with hypothyroidism exhibited the least gut microbial abundance. One study in 2018, which evaluated the gut microbiota composition by using 16S ribosomal RNA gene sequencing in 28 patients with HT and 16 matched healthy controls, displayed that similar levels of bacterial richness and diversity were observed in HT patients and healthy controls [23]. There exists some differences between the two studies. In our study, we divided patients with HT into two groups according to the thyroid function, namely, euthyroidism and hypothyroidism. Among the factors that might influence the stability of gut microbiota, triiodothyronine (T3) is regarded as one of the most important regulators of the development and differentiation of epithelial cells of intestinal mucosa. In addition, variation in thyroid function can affect the gut microbiota [21]. T3 is considered among the most important regulators of the development and differentiation of epithelial cells of intestinal mucosa [24, 25]. Therefore, the difference in the thyroid function of HT patients may contribute to the inconsistency.

In our study, HT patients with euthyroidism have more *Lachnospiraceae_incertae_sedis*, *Lactonifactor*, *Alistipes*, and *Subdoligranulum*, while HT with hypothyroidism have more *Phascolarctobacterium*. Although there is some difference in the gut microbiota composition between healthy controls and HT patients with euthyroidism, the relevant metabolic pathways identified by KEGG pathway analysis are almost similar. Moreover, our analysis demonstrated that *Phascolarctobacterium* is involved in negative regulation on most pathways, such as cellular process, environmental information processing, genetic information processing, and metabolism, which is obviously different from those in healthy controls and HT with euthyroidism. Phascolarctobacterium can produce short-chain fatty acids, including acetate and propionate, and is reported to be associated with the metabolic state and mood of the host [26]. As we know, environmental stress may be the second attack on the basis of the genetic predisposition. Accordingly, we speculated that the change in gut microbiota composition, particularly the increases in *Phascolarctobacterium*, may be associated with the thyroid function status in HT patients.

In our study, we examined the gut microbiota composition in HT patients with different thyroid function status, which was one of the important advantages in our study. Moreover, participants in our study were all females, which might avoid the influence of gender differences. However, there were some limitations in our study. First, this was a single-center study involving a relatively small number of participants, and a large research with multicenter designs would be necessary to validate the relationship. Second, age and the course of HT among the three groups were not well matched, which may influence the data.

In summary, our study demonstrated the altered gut microbiota composition in HT patients with different thyroid function status. Moreover, *Phascolarctobacterium* may be involved in the progression of HT in humans. However, further studies are needed to confirm the findings of the present study and to elucidate the possible underlying mechanisms.

## Figures and Tables

**Table 1:** The clinical characteristics of the participants.

	CON (*n* = 34)	HTN (*n* = 45)	HTH (*n* = 18)	*P* value
Age (years)	29.6 ± 0.6	34.6 ± 1.0^*∗*^	36.3 ± 2.1^*∗*#^	0.0003
BMI (kg/m^2^)	21.4 ± 0.6	22.2 ± 0.5	23.7 ± 0.9	0.0674
Course (months)	0	13.6 ± 2.7^*∗*^	29.7 ± 10.7^*∗*^	0.0001
Levothyroxine use (*n*, %)	0	13 (30%)	13 (72%)	0.0001
TT3 (ng/mL)	1.1 ± 0.02	1.01 ± 0.02	1.10 ± 0.05	0.0812
TT4 (*µ*g/dL)	7.37 ± 0.24	7.15 ± 0.16	6.36 ± 0.26^*∗*^	0.0169
FT3 (pg/mL)	2.91 ± 0.06	2.94 ± 0.05	3.08 ± 0.07	0.2013
FT4 (ng/dL)	1.23 ± 0.03	1.26 ± 0.03	1.06 ± 0.05^*∗*#^	0.0002
TSH (*µ*IU/mL)	1.87 ± 0.16	2.19 ± 0.12	12.70 ± 4.66^*∗*#^	0.0001
A-TG > 115U/mL (*n*/total)	0/31	41/43	11/14	<0.0001
A-TPO > 34 U/mL (*n*/total)	0/33	28/43	9/14	<0.0001

Data are shown as mean ± SEM or number (%). CON, healthy controls; HTN, patients bearing Hashimoto's thyroiditis with euthyroidism; HTH, HT patients with hypothyroidism; BMI, body mass index; TT3, total triiodothyronine; TT4, total thyroxine; FT3, free triiodothyronine; FT4, free thyroxine; TSH, thyroid-stimulating hormone; A-TG, thyroglobulin antibody; A-TPO, thyroperoxidase antibody. ∗Significant difference compared to the CON group. # Significant difference compared with the HTN group.

## Data Availability

The data used to support the findings of this study are available from the corresponding author upon request.
